# Unique Features and Anti-microbial Targeting of Folate- and Flavin-Dependent Methyltransferases Required for Accurate Maintenance of Genetic Information

**DOI:** 10.3389/fmicb.2018.00918

**Published:** 2018-05-09

**Authors:** Hannu Myllykallio, Pierre Sournia, Alice Heliou, Ursula Liebl

**Affiliations:** ^1^Laboratoire d’Optique et Biosciences, Ecole Polytechnique, Centre National de la Recherche Scientifique, Institut National de la Santé et de la Recherche Médicale, Université Paris-Saclay, Palaiseau, France; ^2^Laboratoire d’Informatique de l’École Polytechnique, Ecole Polytechnique, Centre National de la Recherche Scientifique, Université Paris-Saclay, Palaiseau, France

**Keywords:** thymidylate synthesis, RNA modification, flavoproteins, folate dependent methylation, Anti-bacterial Agents, bacterial virulence, *Helicobacter pylori*, *Mycobacterium tuberculosis*

## Abstract

Comparative genome analyses have led to the discovery and characterization of novel flavin- and folate-dependent methyltransferases that mainly function in DNA precursor synthesis and post-transcriptional RNA modification by forming (ribo) thymidylate and its derivatives. Here we discuss the recent literature on the novel mechanistic features of these enzymes sometimes referred to as “uracil methyltransferases,” albeit we prefer to refer to them as (ribo) thymidylate synthases. These enzyme families attest to the convergent evolution of nucleic acid methylation. Special focus is given to describing the unique characteristics of these flavin- and folate-dependent enzymes that have emerged as new models for studying the non-canonical roles of reduced flavin co-factors (FADH_2_) in relaying carbon atoms between enzyme substrates. This ancient enzymatic methylation mechanism with a very wide phylogenetic distribution may be more commonly used for biological methylation reactions than previously anticipated. This notion is exemplified by the recent discovery of additional substrates for these enzymes. Moreover, similar reaction mechanisms can be reversed by demethylases, which remove methyl groups e.g., from human histones. Future work is now required to address whether the use of different methyl donors facilitates the regulation of distinct methylation reactions in the cell. It will also be of great interest to address whether the low activity flavin-dependent thymidylate synthases ThyX represent ancestral enzymes that were eventually replaced by the more active thymidylate synthases of the ThyA family to facilitate the maintenance of larger genomes in fast-growing microbes. Moreover, we discuss the recent efforts from several laboratories to identify selective anti-microbial compounds that target flavin-dependent thymidylate synthase ThyX. Altogether we underline how the discovery of the alternative flavoproteins required for methylation of DNA and/or RNA nucleotides, in addition to providing novel targets for antibiotics, has provided new insight into microbial physiology and virulence.

## Introduction

**FIGURE 1:**
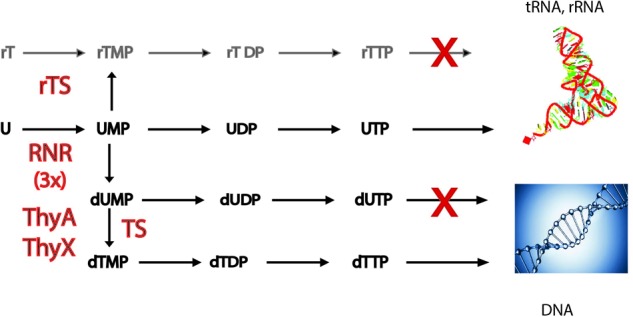
Simplified scheme of the metabolic pathways required for converting ribonucleotides to deoxyribonucleotides. A pathway dependent on a recently described ribothymidylate synthase [rTS (Chen et al., 2016)], methylating UMP to free ribothymidylate (rTMP), would be counter-selected, as systematic methylation of structured RNAs would render them non-functional. Three evolutionarily related families of ribonucleotide reductases (RNR) exist that convert ribonucleotides (A, U, C, and G) to corresponding deoxynucleotides (not discussed here). Thymidylate synthases (TS) convert dUMP to dTMP. The two TS families, ThyA and the flavoprotein ThyX, have distinct evolutionary origins. Misincorporation of dUTP by DNA polymerases into the chromosome is counter-selected, as it would result in an increased mutagenesis rate. For details, see the main text. Incorporation of rTMP to tRNA and rRNA, as well as incorporation of dTMP to DNA are indicated. 3x refers to the three homologous families of RNRs. rTDP, ribothymidine diphosphate; rTTP, ribothymidine triphosphate.

Methylation at the C5 position of the uracil of nucleotides occurs at high frequency per cell division not only during DNA biosynthesis, but also during post-transcriptional RNA modification reactions (see below). The establishment of DNA as genetic material, found nowadays in all free-living cellular organisms and many viruses, required the invention of several enzymatic activities, including not only those required for the conversion of RNA precursors (ribonucleotides) to DNA precursors (deoxyribonucleotides), but also of enzymes capable of methylating deoxyuridylate (dUMP) to deoxythymidylate (dTMP) (Poole et al., 2001). Modern-day metabolic pathways for DNA precursor synthesis provide important clues for understanding how the RNA/DNA transition may have taken place [**Figure 1** (Forterre, 2006)]. The first key step required for the conversion of ribonucleotides to DNA precursors is performed by ribonucleotide reductases (RNRs) that form deoxyribonucleotides via the reduction of ribonucleotides (Torrents, 2014). Three different classes of ribonucleotide reductases exist in present-day organisms. They differ regarding their ways of producing and storing the catalytically necessary cysteinyl radical and are thought to be originating from one common ancestor of modern-day RNRs (Lundin et al., 2015). The homology of the three RNR families was revealed by structural work showing a conserved fold and the conservation of key residues. Thus, conversion of ribonucleotides to deoxyribonucleotides is an excellent example of *divergent evolution*, where the evolution of an ancestral protein led to three different RNR classes with more specialized mechanisms and functions, distinctively used under different growth conditions (Jonna et al., 2015).

The other important difference between DNA and RNA is the systematic presence of deoxythymidine (dT), and not of deoxyuridine (dU), in the DNA of cellular organisms. However, it is well known that some bacteriophages can contain significant amounts of uracil bases in their DNA genomes (Takahashi and Marmur, 1963). Even in the case of some retroviruses like HIV, the reverse transcribed genome equivalent is heavily uracilated protecting it from autointegration (Yan et al., 2011). However, ribothymidine (rT) is also found at specific positions in RNA molecules, where it plays a structural role. The postulated replacement of dU with dT in DNA by thymidylate synthases (TS) allowed the detection of mutations arising from frequently occurring cytosine deamination (Poole et al., 2001). TS are required for *de novo* dTMP synthesis by catalyzing dTMP formation via dUMP methylation at the C5 position of the uracil ring. Two principal TS families are known, ThyA (EC 2.1.1.45) and ThyX (EC 2.1.1.148), which differ in their structures and catalytic mechanisms and are largely exclusive (Hardy et al., 1987; Carreras and Santi, 1995; Myllykallio et al., 2002). Although in modern-day organisms only *free* dUMP is methylated by TS (**Figure 1**), the recent discovery of thymidylate synthase paralogs, capable of methylating the RNA precursors uridine monophosphate (UMP) or cytidine monophosphate (CMP), raised the possibility of free ribothymidylate (rTMP) occuring during DNA precursor synthesis (Chen et al., 2016). However, production of rTMP by ribothymidylate synthases [rTS (**Figure 1**)] and its incorporation to structural RNA molecules would be counter-selected [doctoral thesis, (Sournia, 2016)], since systematic methylation of its base would destabilize tRNA and rRNA structures, thus rendering them non-functional. Note also that incorporation of dUTP into DNA is prevented by dUTPase (dUTP pyrophosphatase) and/or mutagenic dUs are removed from DNA by base excision repair, as they have potential to stall DNA replication forks (Nyiri and Vertessy, 2017). The combined action of these mechanisms keeps DNA uracil-free and thus increases the fidelity of DNA replication.

## Evolutionary Convergence and Mechanistic Diversity of Thymidylate Forming Enzymes

The first enzyme found to catalyze uracil methylation of a nucleotide was thymidylate synthase ThyA (EC 2.1.1.45), encoded by *thyA* in most Prokarya and by the TYMS gene in humans (Carreras and Santi, 1995; Myllykallio et al., 2003). In early work it was demonstrated that ThyA converts 2′-deoxyuridine-5′-monophosphate [dUMP] to the essential DNA precursor 2′-deoxythymidine-5′-monophosphate [dTMP]. Homodimeric ThyA enzymes catalyze the direct transfer of the methylene group from methylene-5,6,7,8-tetrahydrofolate (CH_2_H_4_folate) to dUMP, which is subsequently reduced by H_4_folate to form a methyl group. This reaction results in the formation of dihydrofolate (H_2_folate) that is recycled back to tetrahydrofolate (H_4_folate) by dihydrofolate reductases (Carreras and Santi, 1995). Independently, an S-adenosyl-l-methionine (SAM)-dependent ribothymidylate synthase was discovered in *Escherichia coli* that is called TrmA in bacterial systems (Bjork, 1975; Bjork and Neidhardt, 1975; Ny and Bjork, 1980). TrmA enzymes (E.C.2.1.1.35) methylate C5 of a uridine that is found at position 54 of the T-Psi loop of almost all bacterial functional tRNAs. However, in the majority of archaeal tRNAs, position 54 corresponds to pseudouridines (or its derivatives), although in some archaea U54 is modified to m5U at this position (Phillips and de Crécy-Lagard, 2011; Spenkuch et al., 2014). Differently from ThyA, TrmA *directly* transfers an activated methyl group from SAM to C5 of U54 in tRNA, thus stabilizing its L-shaped conformation and facilitating the decoding of mRNA on the ribosome. We also would like to point out that the eukaryotic (*Saccharomyces cerevisiae*) counterpart of TrmA is called Trm2 (Nordlund et al., 2000). In addition, at least two other SAM-dependent, homologous bacterial methyltransferases are known: RlmD (Agarwalla et al., 2002, 2004), catalyzing the site-specific formation of m^5^U at position 1939 of 23 rRNA, and RlmC that catalyzes the site-specific formation of m^5^U at position 747 in the same 23s rRNA (Madsen et al., 2003).

For decades ThyA and TrmA were considered the only enzymes capable of (deoxy) ribothymidylate formation. This held true until the discovery of two novel flavoproteins that catalyze the formation of thymidylate or ribothymidylate. The first one discovered, deoxythymidylate synthase ThyX [EC 2.1.1.148 (Myllykallio et al., 2002)], is an essentially prokaryotic enzyme, with only very few eukaryotic representatives known. This homotetrameric flavoenzyme uses methylene from CH_2_H_4_folate and acquires the reducing hydride from nicotinamide adenine dinucleotide phosphate (NADPH) (Myllykallio et al., 2002; Leduc et al., 2004b; Koehn et al., 2009). In the case of the RNA metabolism, a gene encoding a new type of bacterial flavin-dependent tRNA:m^5^U_54_-methylase, dubbed TrmFO, was identified about a decade ago (Urbonavicius et al., 2005, 2007). Similarly to ThyX proteins, the TrmFO (previously Gid) flavoproteins (E.C.2.1.1.74) also use CH_2_H_4_folate as donor of a carbon group that is transferred to the uracil ring. Interestingly, the presence of a tRNA methylation pathway, dependent on the cellular reduced folate pools, was found several decades prior to the molecular identification of TrmFO proteins (Romeo et al., 1974; Delk and Rabinowitz, 1975; Delk et al., 1980).

Strikingly, despite all these methylases forming an identical reaction product, methyluracil if only the nucleobase is considered, the four enzyme families are structurally unrelated and use very different reaction mechanisms. This indicates independent evolutionary origins, thus highlighting how Nature has found several independent chemical solutions for transferring a methyl group to a uracil ring (**Table 1**). The observed mechanistic versatility of the DNA/RNA modification machinery thus provides an excellent example of *evolutionary convergence*. An additional example of this versatility are MnmE and MnmG (previously GidA) proteins that form a complex for forming methyluridine derivatives at tRNA wobble positions (Moukadiri et al., 2009; Armengod et al., 2014; Moukadiri et al., 2014). Interestingly, both TrmFO and the MnmEG (GidA) complex are flavin- and folate-dependent tRNA modification enzymes that use structurally similar FAD binding domains (Urbonavicius et al., 2005).

**Table 1:** Overview of the mechanistic diversity of (ribo) thymidylate forming enzymes without structural or sequence similarity.

	ThyA (61%^a^)	ThyX (40%)	TrmFO^b^ (31%)	TrmA (83%)
Structural Fold/ superfamily	TS_pyrimidine_HMase	ThyX	NADB_Rossman	AdoMet_MTase
Substrate	dUMP	dUMP	t-RNA U_54_	t-RNA U_54_
Carbon source	CH_2_H_4_fol	CH_2_H_4_fol	CH_2_H_4_fol	SAM^c^
Substrate activation	Nucleophilic (Cys)	Electrostatic polarization	Nucleophilic (Cys)	Activated methyl group^d^
Reductant	H_4_folate	FAD/ NAD(P)H^e^	FAD/ NAD(P)H	Not required

***^*a*^**Percentage indicating the relative presence of the different enzymes in 711 genomes of the COG database; **^*b*^**Catalytic properties of TrmFO and MnmG/GidA are likely very similar, as suggested by the identification of the catalytically essential conserved cysteine residues participating in substrate activation (Osawa et al., 2009), during synthesis of 5-carboxymethylaminomethyluridine at the tRNA anticodon; **^*c*^**SAM dependent methyltransferases are also involved in cytosine methylation participating in chromatin regulation, gene silencing and protection against DNA cleavage by restriction enzymes; ^*d*^The charged methylsulfonium center of SAM specifically activates the methyl group, thus thermodynamically favoring its direct transfer; **^*e*^**Both, NADH and NADPH can reduce ThyX- and TrmFO-bound FAD co-factor (Myllykallio et al., 2002; Yamagami et al., 2012), see text for details.*

As a substantial portion (up to 7%) of all enzymes in the enzyme classification system is classified as “methyltransferases,” **Table 1** presents only the tip of the iceberg of the biological importance of methylation/alkylation/chemical modification reactions implicating nucleic acids, proteins and small organic molecules found in the cells.

## Unanticipated Flavin-Dependent Methylation Reaction Participates in Biosynthesis of Thymidylate and Nucleoside Antibiotics

Recently, several laboratories have focused their efforts on understanding the reaction mechanisms of ThyX and TrmFO flavoproteins, revealing an unanticipated role for the fully reduced FAD cofactor in the carbon transfer reactions catalyzed by these enzymes.

**FIGURE 2:**
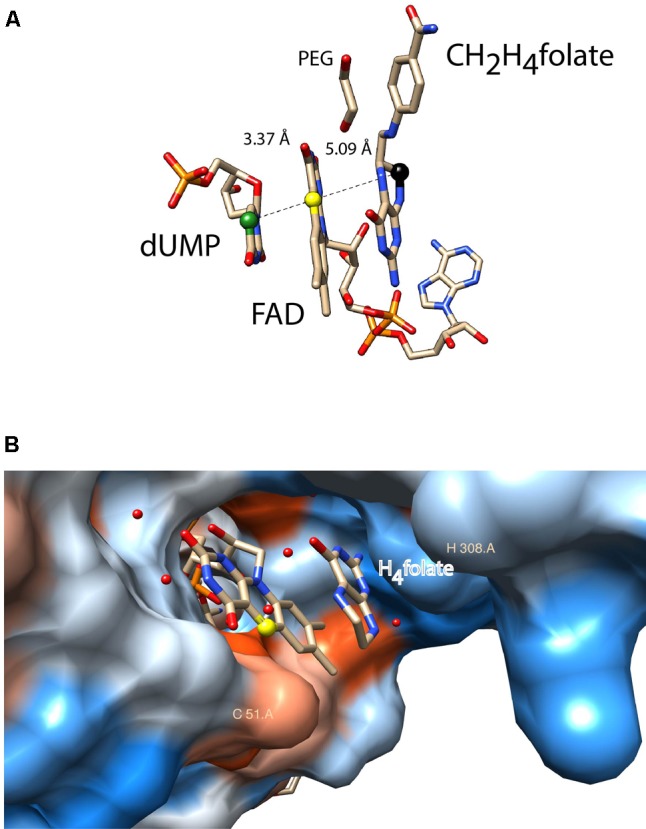
Active site configurations of ThyX and TrmFO proteins as discussed in the text. The figures were made using the program Chimera 1.11.2 **(A)** Binding configuration and stacking interactions of dUMP, FAD, and CH_2_H_4_folate in the active site of the *Thermotoga maritima* ThyX protein (PDB: 4GT9). The proposed one-carbon relay from CH_2_H_4_folate (black ball) via the N5 of the FAD (yellow ball) to the accepting C5 of the uracil ring is depicted. The distances between these atoms are also indicated (Å). The protein backbone and side chains were omitted for reasons of clarity. **(B)** Active site of monomeric *Thermus thermophilus* TrmFO with a fragment of tetrahydrofolate (PDB: 3G5R). The N5 of FAD participates in the carbon transfer reaction analogously to ThyX proteins. The protein surface was colored blue for the hydrophilic residues and orange-red for the hydrophobic ones. The binding site for tRNA has not been experimentally identified. The catalytic residues C51 and H308 (*T. thermophilus* TrmFO numbering) are required for TrmFO-CH_2_-FAD adduct formation, participating in the methylene transfer (see text).

ThyX proteins use dUMP, CH_2_H_4_folate, and NADPH as carbon acceptor, donor, and source of reducing power, respectively. Early steady-state kinetic measurements using a stable viral ThyX enzyme indicated the formation of distinct ternary complexes, with simultaneous binding of at least two substrates to the enzyme (Graziani et al., 2004, 2006). Extensive site-directed mutagenesis and chemical modification experiments identified the location of the very flexible ThyX active site at the interface of three subunits of the ThyX homotetramer, in the vicinity of the redox active N5 atom of the FAD isoalloxazine ring system (Leduc et al., 2004a; Laptenok et al., 2013). Spectroscopic studies of oxidative and reductive half-reactions of ThyX have revealed a complex interplay of the three ThyX substrates at the active site that prevents the unwanted turnover of ThyX proteins with molecular oxygen (Gattis and Palfey, 2005; Wang et al., 2009; Becker et al., 2014). *A priori*, transfer of a methylene group from CH_2_H_4_folate to the dUMP nucleotide could be direct, as has been revealed for the ThyA family of TS (Carreras and Santi, 1995). However, recent mechanistic studies have managed to trap a key reaction intermediate in *Thermotoga maritima* ThyX that was identified as a dUMP-CH_2_-FADH_2_ bridged compound, suggesting that FADH_2_ participates in the transfer of one-carbon units (Mishanina et al., 2016). This notion was also supported by the 5-deazaflavin-reconstituted enzyme being inactive, thus indicating the nucleophilic participation of reduced FAD in the ThyX methylation reaction (Mishanina et al., 2012, 2016). Interestingly, structural data has indicated that both, CH_2_H_4_folate and dUMP are bound in close vicinity (Koehn et al., 2012) at opposite sides of the FAD isoribityl ring system (**Figure 2A**), indicating that (N5) FAD shuttles a methylene group (and not electrons, as in up to 90% of flavoproteins) from one side of the FAD ring to the other during enzymatic formation of thymidylate (Mishanina et al., 2016). A further key mechanistic difference between ThyA and ThyX is that the latter uses an electrostatic, and not covalent, activation of the methylene-receiving C5 of dUMP (Stull et al., 2016). This observation convincingly explains why early efforts to identify nucleophilic amino acid residues of ThyX proteins involved in substrate activation were unsuccessful.

Strikingly, recent studies have demonstrated that some *Streptomyces* species carry two different *thyX* genes. One ThyX paralog, dubbed PolB, is involved in the synthesis of polyoxin nucleoside antibiotics in *Streptomyces viridochromogenes*. This is not surprising, as the C5 modifications occurring during polyoxin biosynthesis are similar to reactions catalyzed by ThyX enzymes (Chen et al., 2016). As mentioned above, *in vitro* these neofunctionalized paralogs have partially lost their original substrate specificity as they can methylate *both* dUMP and UMP in solution, indicating that a single protein carries *both* thymidylate and ribothymidylate synthase activities. These observations have revealed unexpected links between pyrimidine and nucleoside antibiotic metabolisms that are further supported by identification of *thyA* paralogs that also participate in the biosynthesis of nucleoside antibiotics (Zhao et al., 2016). These examples illustrate how DNA metabolic enzymes have evolved toward a functional role in secondary nucleoside biosynthesis acting as anti-microbial compounds.

As mentioned above, similarly to ThyX, TrmFO is also an FAD-dependent enzyme that transfers the methylene group from CH_2_H_4_folate to the C5 of the uracil ring (U54 of tRNAs). As isolated, *Bacillus subtilis* TrmFO is a mixture of different spectroscopically distinguishable species, one of which is capable of tRNA methylation without added substrates (Urbonavicius et al., 2005; Hamdane et al., 2011a,b). Prior to the identification of a similar intermediate in ThyX (see above), one of these spectroscopic forms was characterized as a covalent enzyme-methylene-FAD adduct by mass spectrometry (Hamdane et al., 2012). The optical absorption spectrum of this adduct has a broad maximum at 360 nm, which is fully consistent with an alkylated FAD that is covalently bound to the enzyme via a cysteine residue (Cys51 in *Thermus thermophilus* TrmFO, **Figure 2B**), as was demonstrated via mutagenesis studies (Hamdane et al., 2011a). These results indicate that the catalytic cysteine is required for formation of a reaction intermediate, where CH_2_ is transferred to tRNA using FAD as a methylating agent. This unique flavin-dependent tRNA-methylation can be activated at low pH, likely corresponding to a protonation of the catalytic cysteine (Hamdane et al., 2013). Note also that the actual methylation agent is likely not the above-described intermediate, but rather its highly reactive iminium form [(FADH(N5) = CH_2_]^+^ (Hamdane et al., 2012). Structural data for *T. thermophilus* TrmFO indicates binding of a H_4_folate molecule in the vicinity of the FAD isoribityl ring (**Figure 2**); fully consistent with the proposed carbon transfer mechanism (Nishimasu et al., 2009; Yamagami et al., 2012). The RNA binding site of TrmFO enzymes is not known, but tRNA binding is likely to bring about important configurational changes, similar to what is observed for TrmA. Although the location of the accepting uracil base is unknown, a second catalytic cysteine (not shown in **Figure 2**) is implicated in the activation of uridine via covalent catalysis, as a mini-tRNA substrate becomes covalently attached if it carries fluorine at the C5 position of U54 of tRNA (Hamdane et al., 2011a). Moreover, our very recent studies on *T. thermophilus* TrmFO allowed the real-time detection of a protonated tyrosyl radical cation as a short lived redox intermediate (Nag et al., 2017).

Altogether, these exciting recent developments suggest that ThyX and TrmFO proteins use a unique carbon-relay mechanism where the reduced FAD plays a key role as methylating agent, but that the two enzymes differ fundamentally in terms of how the receiving substrates (U54 or dUMP) are “activated” by the different enzymes in order to accept the carbon group.

## Relative Frequency and Phylogenetic Distribution of Thymidylate Forming Enzymes

**FIGURE 3:**
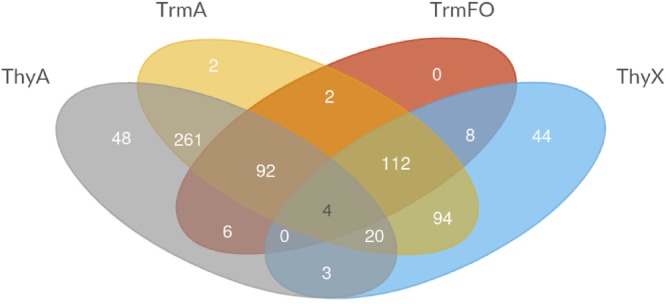
Venn diagram depicting the different combinations of thymidylate-forming enzymes ThyA, ThyX, TrmA, and TrmFO in the species included in the COG database (Galperin et al., 2017).

**Table 2** demonstrates the presence and absence of the different (ribo) TS and related enzymes in the major archaeal and bacterial groups, as reported in the COG database based upon 711 species (Galperin et al., 2017). Note the substantial presence of all four enzymes in living organisms, as even the least common TrmFO family is found in at least 30% of the analyzed COG genomes (**Table 2**). To investigate whether all the possible combinations of thymidylate synthase-forming enzymes are found in Nature, we constructed a Venn diagram indicating the presence of the different enzymes. This analysis revealed that the most often observed combinations carry thymidylate synthase ThyA or ThyX in a combination with TrmA, whereas some combinations ((ThyA^+^ ThyX^+^ TrmA^-^ TrmFO^+^ and ThyA^-^ ThyX^-^ TrmA^-^TrmFO^+^) are less frequent (**Figure 3**). In very few cases, Life *without* TS appears feasible at first sight, but in-depth analysis reveals that all these species carry the *tdk* gene encoding thymidine kinase, required for the salvage and/or intracellular recycling of thymine and/or thymidine. This is fully compatible with the essentiality of TS ThyA and ThyX in virtually all analyzed species. The apparent absence of tRNA and/or rRNA uracil methylation (m^5^U at position 54 of tRNA, as well as at positions 747 and 1939 of 23 rRNA) in 13% of the analyzed genomes seems curious, but it is well known that methyltransferase activities of TrmA (Bjork and Isaksson, 1970) and TrmFO (Urbonavicius et al., 2005) are dispensable for growth under optimal growth conditions. Recent studies have indicated that *T. thermophilus* TrmFO regulates the network of many other tRNA modifications (e.g., 2′-O-methylguanosine and N(1)–methyladenosine), particularly under suboptimal growth conditions (Yamagami et al., 2016). Under these conditions, induced by the relatively low growth temperature, deletion of *trmFO* leads to defects in protein synthesis, thus influencing the cell metabolism at a global level. Moreover, the consumption of CH_2_H_4_THF by TrmFO limits the rate of dTMP synthesis by ThyX, thus resulting in slow growth under nutrient-poor conditions (Yamagami et al., 2018). This implies that (ribo) thymidylate-forming enzymes may influence differentially the folate cycles in actively dividing cells, as we have previously proposed (Leduc et al., 2007).

**Table 2:** Presence and absence of thymidylate-forming^a^ enzymes in the COG database in different microbial phyla.

	ThyX	TrmFO (Gid)^a^	MnmG (GidA)	ThyA	TrmA family
Group^b^ (number of genomes)					
**Archaea**		
Crenarchaeota (21)	20	0	0	2	6
Euryarchaeota (56)	15	0	0	40	26
Thaumarchaeota (4)	4	0	0	0	0
Other archaea (2)	2	0	0	0	2
**Bacteria**		
Acidobacteria (6)	6	6	6	0	6
Actinobacteria (74)	40	8	0	47	68
Aquificiaea (8)	8	8	8	0	8
Bacteroides (55)	0	1	55	53	49
Chlorobi (5)	5	0	5	1	5
Chlamydiae (6)	6	0	6	0	6
Chloroflexi (9)	9	2	4	2	8
Cyanobacteria (31)	23	25	31	8	31
Deinococcus/ Thermus (6)	4	6	6	2	6
**Firmicutes**		
Bacilli (33)	3	23	30	31	33
Clostridia (49)	43	32	44	10	49
Mollicutes (10)	0	3	10	9	1
Other Firmicutes (6)	3	5	5	3	6
Fusobacteria (5)	0	2	5	3	5
Planctomycetes (6)	1	0	6	5	3
**Proteobacteria**		
Alpha (75)	28	55	75	48	59
Beta (52)	0	0	52	49	46
Delta (28)	17	23	26	11	25
Epsilon (11)	10	0	11	1	10
Gamma (103)	2	0	102	96	90
Spirochetes (7)	6	0	7	1	5
Synergistetes (5)	4	4	4	0	4
Thermotogae (7)	7	5	7	0	7
Other Bacteria (31)	19	16	25	12	23
Total (711 genomes)	285 (40%)	224 (31%)	530 (75%)	434 (61 %)	587 (83%)

*^*a*^MnmG, a tRNA uridine 5-carboxymethylaminomethyl modification enzyme was included in the table due to its structural similarity to TrmFO enzymes. These enzymes are also found in Eukarya, particularly in ascomycetes; ^*b*^Different bacterial groups with the number of analyzed genomes indicated in parentheses. For detailed information on the different enzyme families, see the main text.*

Note also that TS ThyA and ThyX and RNA methyltransferase TrmA have sporadic phylogenetic distributions and participate commonly in lateral gene transfer (LTG) events that have been quantitatively studied using a probabilistic evolutionary model developed for non-homologous gene displacements accounting for gene loss and gain (Stern et al., 2010). The obtained results indicated that the phylogenetic distributions of ThyA and ThyX are greatly influenced by LTG between bacteria, archaea and their viruses. Moreover, at least mycobacteria and the Crenarchaeon *Ignisphaera aggregans* carry both ThyA and ThyX (but not RNA uracil methylases), which may correspond to a transition stage in LTG (**Figure 3**). **Figure 3** revealed four species (*Beijerinckia indica, Bacillus anthracis, Candidatus Puniceispirillum marinum*, and *Macrococcus caseolyticus*) that have all four pathways (including two TS), exemplifying in part the frequent LGT of thymidylate synthase genes. LGT has also shaped the evolution of TrmA RNA methyltransferases, as some archaea, such as *Nanoarchaea* and *Thermococcales*, likely acquired TrmA-like genes through an ancient horizontal gene transfer event, followed by a duplication event and change of substrate specificity from rRNA methylase to tRNA (Urbonavicius et al., 2008; Auxilien et al., 2011). On the other hand, the phylogenetic distribution of TrmFO proteins is more congruent with the bacterial phylogenies based upon the ribosomal RNA sequences (Urbonavicius et al., 2007), albeit TrmFO paralogs are known to methylate not only tRNA, but also rRNA molecules (Lartigue et al., 2014). Note also that despite TrmFO (and MnmG) proteins have evolved from classic Rossman fold flavoproteins (Urbonavicius et al., 2005; Nishimasu et al., 2009), the evolutionary origin of ThyX proteins remains elusive. However, it is tempting to speculate that ThyX proteins are ancient, as they are frequently found in both archaea and bacteria (**Table 2**). If this is the case, thymidylate synthase ThyX could represent an ancestral thymidylate synthase that was eventually replaced by the more active TS of the ThyA family to facilitate the maintenance of larger genomes in fast-growing microbes. Indeed, the catalytic efficiency estimated using k_cat_/K_m_ values of ThyX proteins is approximately 10–20 times less than what is typically measured for thymidylate synthase ThyA (Escartin et al., 2008).

Note also that mechanistic and evolutionary aspects of (ribo) TS have also been previously discussed (Sournia, 2016).

## Ecological Genomics of Thymidylate Synthases

**FIGURE 4:**
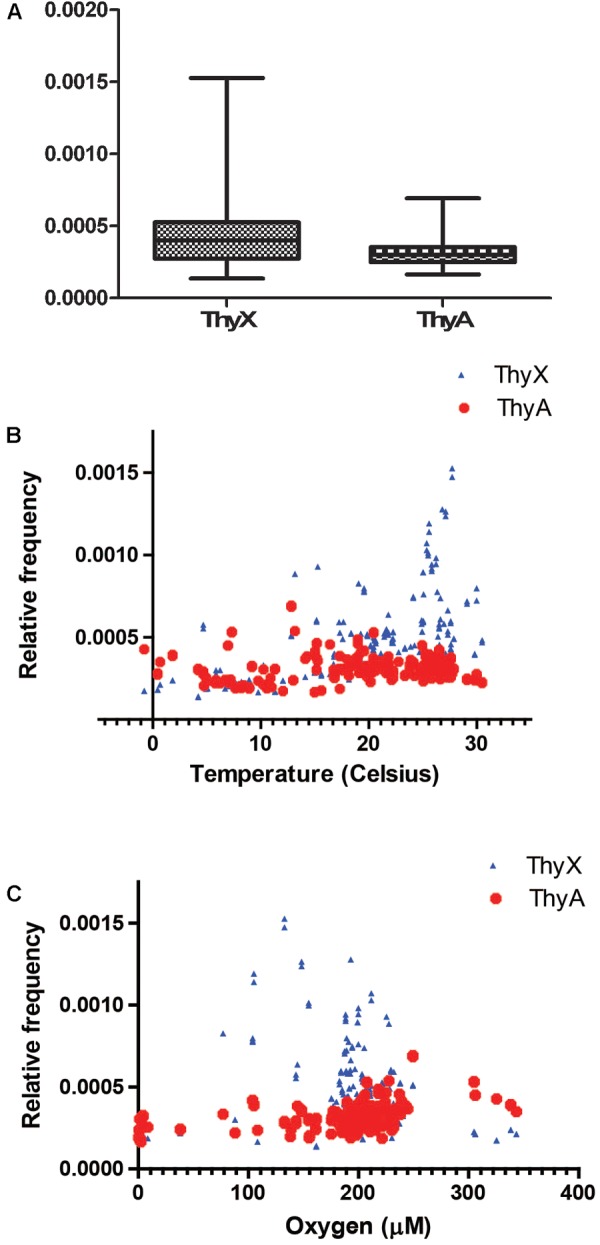
**(A)** Relative frequencies of ThyX and ThyA sequences in 243 Tara Ocean samples (Sunagawa et al., 2015). For ThyX and ThyA, 5,205,730 and 3,571,073 individual reads were analyzed, respectively. Significantly different means of the relative frequencies for ThyX and ThyA sequences were 0.00044 and 0.0003 (*p* < 0.05, unpaired *t*-test with Welch’s correction without assuming equal variance). **(B,C)** Relative frequencies of ThyX and ThyA sequences as a function of the isolation temperature **(B)** and oxygen concentration **(C)** of Tara Ocean samples.

To address whether ecology could drive lateral gene exchange of TS and/or whether environmental conditions could differentially modulate their enzymatic activities, we benefited from the quantitative DNA sequencing data made available by the Tara Ocean project^1^, destined to obtain structural and functional insight into the global ocean microbiome. Altogether, 8776803 microbial or viral reads classified either as “thymidylate synthase ThyX or ThyA” were identified from more than 240 environmental sampling sites. As 59.3% of these reads were classified as “ThyX” and medians of relative frequencies of ThyX sequences in the different samples were significantly higher than those of ThyA (*p*-value < 0.05), species carrying ThyX are abundant in these ocean metagenome samples (**Figure 4A**). As the temperature and oxygen are the major factors influencing the ocean microbial community composition (Sunagawa et al., 2015), we plotted the relative frequencies of ThyX and ThyA sequences as a function of the reported isolation temperatures and oxygen concentration (**Figures 4B,C**), as well as the isolation depth (Supplementary Figure 1). These plots show the abundant presence of thymidylate synthase ThyX in the epipelagic zone where temperature ranges from 25 to 30 and isolation depth is less than 100 meters. This analysis reveals that the spatial distribution of ThyX and ThyA carrying species in these ocean samples is non-random, suggesting that environmental constraints may influence non-homologous TS in different ways. To further explain this observation, we stress that the bacterial phyla that dominate the epipelagic zone include several slow-growing microbial species such as the SAR11 clade, cyanobacteria (*Prochlorococcus* and *Synechococcus*), *Deferribacteraceae* and *Thaumarchaeota* that have undergone massive genome streamlining under nutrient limiting conditions (Giovannoni et al., 2014). All these bacteria are expected to use ThyX that is preferentially found in slow-growing/replicating bacteria with small genomes (Escartin et al., 2008). We also stress that many ThyX proteins have built-in mechanisms for preventing unwanted turnover of the reduced FAD co-factor with molecular oxygen (Becker et al., 2014). Consequently, the presence of oxygen does not necessarily limit the thymidylate synthase activity of ThyX proteins in these oxygenic samples.

## Mutations in Folate- and Flavin-Dependent Methyltransferases Modulate Bacterial Virulence

Strikingly, recent genome-wide sequencing studies have also implicated the thymidylate metabolism as virulence factor. This is exemplified for instance by the fact that many *Staphylococcus aureus* small colony variants are thymidine autotrophs that are associated with persistent antibiotics resistant infections in hospitals (Kahl et al., 2016). It is thus not surprising that frameshift mutations within *Leptospira interrogans thyX* attenuate virulence in this spirochete, a causative agent of leptospirosis (Lehmann et al., 2013). These studies also revealed substantial *in vivo* upregulation of *L. interrogans thyX* when compared with *in vitro* cultured bacterial cells. Moreover, replacement of *thyX* by *thyA*, which encodes a catalytically more efficient thymidylate synthase, thus possibly increasing fitness (Escartin et al., 2008), has been linked to hypervirulence in *Clostridium difficile* (Knetsch et al., 2011). Systematic studies have associated promoter mutations resulting in thymidylate synthase ThyX overexpression in *Mycobacterium tuberculosis* with drug resistance (Zhang et al., 2013). Recently it has also been proposed that mutations occurring during the intra-patient evolution of multi-drug resistant *M. tuberculosis* strains result in up-regulation of ThyX (Merker et al., 2013). Altogether, these observations indicate that the thymidylate metabolism is not only required for growth, but also plays a role in shaping virulence and fitness of pathogenic bacteria.

Mutations in the tRNA modification enzyme MnmG/GidA also influence bacterial virulence, presumably because they increase the frequency of a two-base frameshift during translation of mRNA (Shippy and Fadl, 2015; Gao et al., 2016). This led to suggestions that a *gidA* deletion could be used as an attenuated vaccine candidate (Shippy and Fadl, 2012). Another potential use of MnmG/GidA enzymes could be as target for modulating bacterial virulence using chemical compounds. The latter could also be true for TrmFO enzymes, considering their global relevance for the bacterial cell metabolism described above. This approach is nevertheless complicated by the fact that tRNA modification in human mitochondria is dependent on MTO1 enzymes related to GidA (Armengod et al., 2014).

## ThyX Proteins as Targets for New Antimicrobial Compounds

As microbial ThyX proteins have no structural similarity to human thymidylate synthase ThyA, and the active site configurations and catalytic mechanisms of ThyX and ThyA proteins are drastically different, selective inhibition of ThyX is a highly feasible goal. This is of particular interest, as ThyX enzymes are found in many enteric pathogens or bacteria causing zoonotic diseases, including *Helicobacter, Mycobacteria* and *Clostridium* species. Together with their unique biochemical reaction mechanism, these observations make ThyX proteins attractive drug targets and extensive efforts to identify ThyX inhibitors are underway in several laboratories.

To date, several classes of ThyX inhibitors have been discovered. Early efforts of targeting ThyX allowed the identification of two distinct classes of compounds, based on a thiazolidine core, as inhibitors of thymidylate synthase X with submicromolar concentrations (Esra Onen et al., 2008). Activity-based screening of a small library of 2500 chemically diverse molecules identified 1,4-naphthoquinone (NQ) derivatives as specific inhibitors of ThyX proteins (Basta et al., 2012), without affecting human thymidylate synthase. To date, the most interesting of these inhibitors with a clear dose-response against *Helicobacter pylori* have *K*_i_-values in the range of 200–300 nM (Skouloubris et al., 2015). These mechanism-based inhibitors prevent the formation of the catalytically relevant FADH_2_, required for the transfer of one-carbon units, show competitive inhibition with respect to dUMP and a non-competitive inhibition mode with respect to the other substrates. In agreement with these findings, structural data have indicated that the inhibitor binding site considerably overlaps with the dUMP binding pocket (Basta et al., 2012). Analogous inhibitors were recently identified using virtual screening and structural approaches that revealed the first X-ray crystal structure of *Thermatoga maritima* ThyX in complex with a non-substrate analog inhibitor, identifying 1H-imidazo[4,5-d]pyridazine as a scaffold for the development of *Mtb*-ThyX inhibitors (Luciani et al., 2016). Testing the *in vivo* efficacy of identified non-cytotoxic, non-mitotoxic 2-OH-1,4-NQ inhibitors in a mouse model for *H. pylori* infections identified tight-binding ThyX inhibitors that were tolerated in mice and can be associated with a modest effect in reducing the number of colonizing bacteria, thus providing proof-of-concept that targeting ThyX enzymes is a highly feasible strategy for the development of therapies against *H. pylori* and other ThyX-dependent pathogenic bacteria (Skouloubris et al., 2015).

Differently from the large majority of bacteria, where the distribution of *thyX* and *thyA* is mutually exclusive, mycobacteria contain both, *thyA* and *thyX* genes. While both TS are expressed in *M. tuberculosis*, mutational studies have shown that *thyX* is essential, further confirming the enzyme as an attractive drug target (Fivian-Hughes et al., 2012). A 5-substituted dUMP analog was identified that lacks activity against mycobacterial ThyA and displays an IC_50_ value against mycobacterial ThyX of 0.91 μM (Kogler et al., 2011). In a continuation of these studies, 5-substituted 6-aza-dUMP derivatives were found to exhibit weak ThyX inhibitory activity (Kogler et al., 2012). Compounds with a 5-alkynyl uracil moiety coupled to an acyclic nucleoside phosphonate (ANP) showed modest inhibition of ThyX to date, but lend themselves as starting point for the development of more potent compounds (Parchina et al., 2013). Several phosphoroamidate derivatives of *N*-(3-(5-(2′-deoxyuridine-5′-monophosphate))prop-2-ynyl) octanamide were designed in order to improve permeability through the mycobacterial cell wall. Biological tests of these prodrugs showed antimycobacterial activity against *M. tuberculosis* H37Rv and *M. bovis* BCG, suggesting that they were able to penetrate the mycobacterial cell wall, thus liberating the monophosphate intracellularly and targeting ThyX (McGuigan et al., 2014). Further systematic structure-activity relationship analyses combined with docking studies revealed 5-undecyloxymethyl-2′-deoxyuridine 5′-monophosphate, displaying an IC50 value against ThyX of 8.32 μM and lacking activity against ThyA (Alexandrova et al., 2015). Finally, using a combination of chemoinformatics and *in vitro* screening, new *Mtb* ThyX inhibitors, 2-chloro-3-(4-methanesulfonylpiperazin-1-yl)-1,4-dihydronaphthalene-1,4-dione) and idebenone were identified that show modest whole-cell activity. As idebenone has already passed clinical trials for unrelated uses and targets at least partially ThyX in living cells, this is very encouraging for the further development of ThyX inhibitors toward biomedical applications (Djaout et al., 2016). A recent high-throughput screening campaign of 40,000 compounds performed for identification of new ThyX inhibitors (Abu El Asrar et al., 2017) lead to the discovery of new *M. tuberculosis* ThyX inhibitors with a novel action mode.

It is obvious that ThyX inhibitors that exploit the novel reaction mechanism of ThyX proteins are expected to have a large impact, as several important human pathogens, including *Helicobacter, Mycobacteria, Chlamydia* or *Rickettsia* species, and many causative agents of bacterial neglected diseases rely on ThyX for *de novo* dTMP synthesis.

## Conclusion and Perspectives

Here we have described how all roads lead from U to T, with a special focus on two flavin-dependent enzymes, ThyX and TrmFO, which are required for DNA precursor synthesis and post-transcriptional modification of RNA, respectively. As ThyX and TrmFO enzymes are evolutionarily unrelated they also provide an excellent example how Nature has repeatedly modified the microenvironment of the reduced FAD co-factor to modulate its chemical reactivity, thus facilitating carbon transfer. From a mechanistic point of view, these enzymes are emerging as unique models for the relay of a methylene group by using the FAD cofactor as covalent catalyst for modifying not only nucleic acids and their precursors, but also nucleoside antibiotics. This catalytic strategy might therefore be more common than previously anticipated and clearly deserves further studies. Future work is thus required to address how common the non-canonical roles of FADH_2_ are in carbon transfer reactions involving secondary metabolites as receiving substrate. It is also of interest to note that human histone demethylases catalyze the inverse reaction by removing methyl groups using FAD as a cofactor and thus influence epigenetic regulation (Shi et al., 2004; Dimitrova et al., 2015).

Based upon complete genome sequences and quantitative metagenomics data, we further propose that functional and/or ecological constraints may influence the utilization of these enzymes and LTG events implicating the corresponding genes. Moreover, the thymidylate metabolism also provides an excellent example how metabolic enzymes participate in modulating bacterial virulence. As enzymes using a ThyX-like reaction mechanism have not yet been identified in humans, the mechanistic and inhibitory studies discussed here have potential to aid in the development of new anti-microbial compounds. In this respect it is noteworthy that several ThyX inhibitors with anti-microbial activity have been reported, prompting further optimization by exploiting the newly discovered reaction mechanisms in order to provide new antibiotics.

Future work is now needed to address why both, CH_2_H_4_folate- and SAM-dependent nucleic acid methylases do exist in the cell? Does the use of different methyl donors facilitate regulation of the distinct methylation reactions? Finally, it will also be of great interest to address whether the low activity thymidylate synthase ThyX family could correspond to the ancestral enzymes that were replaced by the more active ThyA enzymes during evolution to facilitate the maintenance of large genomes in fast-growing microbes.

## Author Contributions

HM and UL planned and wrote the manuscript. PS and AH contributed ideas, critical analyses, and prepared figures.

## Conflict of Interest Statement

The authors declare that the research was conducted in the absence of any commercial or financial relationships that could be construed as a potential conflict of interest.

## Footnotes

1 https://www.embl.de/tara-oceans/start/
