# Supplementary material for: Unique Features and Anti-microbial Targeting of Folate- and Flavin-Dependent Methyltransferases Required for Accurate Maintenance of Genetic Information
Source: Front Microbiol. 2018 May 9;9:918. doi: 10.3389/fmicb.2018.00918 (PMC5954106; doi:10.3389/fmicb.2018.00918)

Supplementary Figure 1: Relative frequencies of ThyX and ThyA sequences as a function of the isolation depth of Tara Ocean samples. Notice the logarithmic scale (X-axis).

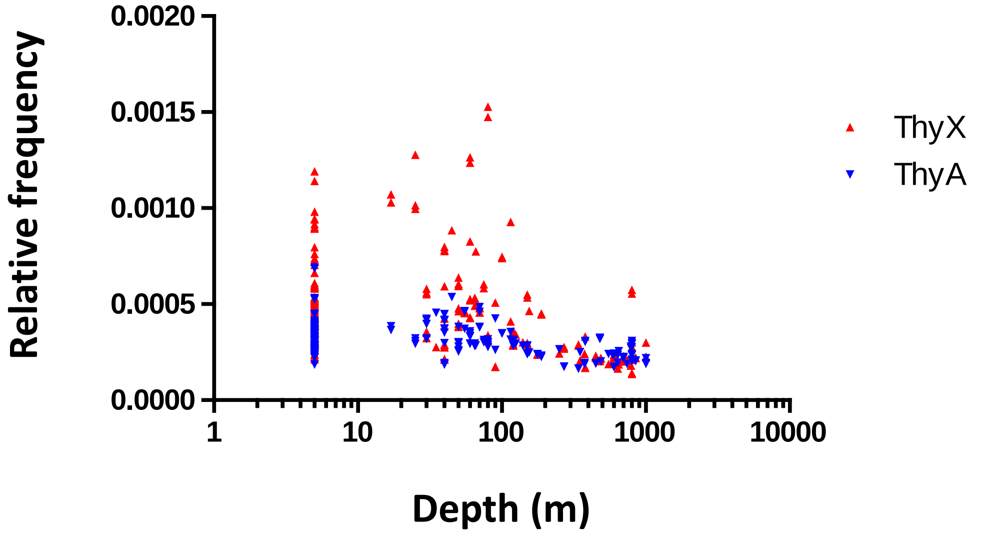

Supplement: Supplementary file 1 [file Image_1.PDF]
